# OGTT-Defined Insulin Resistance in Adults With Schizophrenia and Schizoaffective Disorder: Comparing Clozapine versus other Antipsychotics

**DOI:** 10.1192/j.eurpsy.2026.10532

**Published:** 2026-07-31

**Authors:** C. Esme, E. Van Assche, C. Hohoff, S. Claes, M. De Hert, B. Baune

**Affiliations:** 1 Department of Psychiatry, University of Münster, Münster, Germany; 2 Department of Psychiatry, UPC KU Leuven, Leuven, Belgium; 3 Department of Psychiatry, University of Melbourne; 4 The Florey Institute of Neuroscience and Mental Health,Melbourne Medical School, Melbourne, Australia

## Abstract

**Introduction:**

Antipsychotic-associated metabolic dysfunction and weight gain is a significant concern, particularly raised in the context of clozapin treatment. Insulin resistance (IR) is often named as a facilitator in this relationship. Evidence from oral glucose tolerance tests (OGTTs) to compare clozapine with other antipsychotics is limited and inconsistent. The present study compares IR by OGTT between clozapine-treated patients and non-clozapine-treated patients, with a particular focus on the role of duration of illness (DOI). We expect an increased risk for insulin resistance by OGTT in the patient group receiving clozapine, compared to other antipsychotics.

**Objectives:**

Compare insuline resistance between clozapine and non-clozapine users, taking duration of illness into account.

**Methods:**

A total of 379 individuals with a diagnosis of schizophrenia or schizoaffective disorder (Mage= 34.05; %67.28 Men) were included in our analysis. Sixty-seven patients (Mage= 35.68; 68.66% men) were treated with clozapine at the time of investigation. The other patients (Mage= 33.70; 66.99% men) were treated with risperidon (N = 82), Amisulprid (N = 34), Ariprazol (N = 7), Olanzapine (N = 113), Quetiapine (N = 36), 1st generation antipsychotics (N = 26), or no antipsychotic (N = 14). Insulin resistance was measured with OGTT (MIR= 2.62; SD = 2.12). Mean duration of illness (DOI) was 10.70 years (SD = 9.79). Analyses were performed in R using Anova and linear regression. Analyses were corrected for biological sex and age.

**Results:**

Following adjustment for DOI, age and sex, insulin resistance as measured with OGTT was not statistically significant between patients treated with clozapine and patients treated with other antipsychotics (F(1, 360) = 1.71; p > 0.10). DOI was not associated with IR either (p > 0.10), however clozapine treatment was associated with DOI after adjustment for age and sex (F(1, 362)= 0.146; p< 0.001), with a longer DOI for patients receiving Clozapine.Stratification using a DOI-by-treatment group analysis also did not affect the risk for IR significantly either (p > 0.10).

**Conclusions:**

Our results indicated that clozapine exposure and duration of illness—either independently or in interaction—were not associated with a an elevated risk of OGTT-defined insulin resistance, in comparison to other antipsychotics. The observed difference in illness duration, with a longer duration of illness among clozapine users compared to other patients, is consistent with clozapine’s frequent use as a third-line treatment. Contrary to our initial hypothesis, our data did not demonstrate a higher risk for insulin resistance in patients treated with clozapine, nor in those with a prolonged history of disease.

**Disclosure of Interest:**

None Declared

